# *RsGSTF12* Contributes to Anthocyanin Sequestration in Radish (*Raphanus sativus* L.)

**DOI:** 10.3389/fpls.2022.870202

**Published:** 2022-07-04

**Authors:** Mengyang Niu, Changjian Bao, Jiahui Chen, Wen Zhou, Yueyue Zhang, Xiaoyan Zhang, Nana Su, Jin Cui

**Affiliations:** College of Life Sciences, Nanjing Agricultural University, Nanjing, China

**Keywords:** radish, anthocyanin, glutathione S-transferase, vacuole, sequestration

## Abstract

Anthocyanins are water-soluble plant pigments mainly stored in the plant vacuoles. Glutathione S-transferases (GSTs) are a multifunctional enzyme family, which can regulate substance metabolism and biological and abiotic stresses in plants. However, few reports were focused on the involvement of GSTs in anthocyanin sequestration in red skin radish. Here, we identified a glutathione S-transferase gene *RsGSTF12* that played roles in anthocyanin sequestration in radish. The bioinformatics analysis revealed that RsGSTF12 belonged to the phi (F) class of glutathione S-transferases and showed a high homology with AtGSTF12, followed by AtGSTF11. The subcellular localization assay showed that RsGSTF12 was located in the endoplasmic reticulum and tonoplast. Temporal and spatial gene expression-specific analyses uncovered a strong correlation of *RsGSTF12* with anthocyanin accumulation in radish sprouts. The anthocyanin solubility assay found RsGSTF12 was capable of improving cyanidin water solubility *in vitro*. Transiently expressing *RsGSTF12* in radish cotyledons was able to increase their anthocyanin sequestrations. Furthermore, the functional complementation and overexpression of the *Arabidopsis thaliana tt19* mutant and wild type demonstrated that *RsGSTF12* might play an indispensable role in anthocyanin accumulation in radish. Taken together, we provide compelling evidence that RsGSTF12 functions critically in how anthocyanins are sequestrated in radish, which may enrich our understanding of the mechanism of anthocyanin sequestration.

## Introduction

Anthocyanins, an important kind of secondary metabolites that widely exist in the plant kingdom, are water-soluble plant pigments mainly stored in the vacuole of plant cells that are responsible for giving plants their pink, orange, red, purple, and blue colors (Tsuda, 2018). Anthocyanins can also attract insects for pollination and seed transmission, protect photosynthetic tissues from oxidative damage, protect cells from ultraviolet radiation damage, and confer resistance to pathogens as an antibacterial agent (Harborne and Williams, 2000; Winkel-Shirley, 2001). Another key characteristic of anthocyanins is their antioxidant activity; hence, anthocyanins are indispensable for scavenging free radicals, resisting cancers, inhibiting tumors, and preventing neuronal diseases and cardiovascular diseases (Liu et al., 2018).

Anthocyanins are synthesized *via* the flavonoid biosynthesis pathway, a branch of the phenylpropanoid metabolic pathway (Grotewold, 2006). Anthocyanin biosynthesis is a process catalyzed by enzymes that are encoded by a series of structural genes. Along phenylpropane branches, the following enzymes are catalyzed by multi-step biochemical reactions (Winkel-Shirley, 2001): phenylalanine ammonia-lyase (PAL), cinnamic acid by cinnamate-4-hydroxylase (C4H), 4-coumaric acid CoA ligase (4CL), chalcone synthase (CHS), chalcone isomerase (CHI), flavanone 3-hydroxylase (F3H), flavone 3’-hydroxylase (F3’H), dihydroflavonol 4-reductase (DFR), anthocyanin synthase (ANS), and UDP sugar flavonoid glycosyltransferase (UFGT). The above related enzymes involved in anthocyanin synthesis exist in cell solute, and anthocyanin is transferred to the vacuole after synthesis (Jorgensen et al., 2005; Li et al., 2012). At present, the research on the regulation of anthocyanin synthesis is more thorough (Lin-Wang et al., 2011; Cavallini et al., 2015; He et al., 2020), while the research on the anthocyanin sequestration is still in its infancy (Zhao and Dixon, 2010; Kou et al., 2019). It is generally believed that anthocyanins are synthesized by a series of synthases on the cytoplasmic surface of the endoplasmic reticulum, which makes it very important to study the mechanism of anthocyanins transferred from the endoplasmic reticulum to vacuoles. Presently, there are two hypothetical models of anthocyanin import to vacuoles: (1) *vesicle-trafficking model*—small vesicles filled with anthocyanins can move toward the central vacuole, as observed in grapevine and Arabidopsis (Kitamura et al., 2010; Gomez et al., 2011) and (2) *ligand protein-mediated model*—glutathione S-transferases (GSTs) commonly function as ligandins of anthocyanin to bind different anthocyanin monomers, and then, the anthocyanins are escorted from the cytosol to the tonoplast. After that, anthocyanin is transferred to the vicinity of vacuoles, by moving across the tonoplast through transporters, such as MRP-type ATP-binding cassette (ABC) transporter and the multidrug and toxic compound extrusion (MATE) transporter (e.g., TT12) (Debeaujon et al., 2001; Zhao and Dixon, 2010). Many studies have suggested that vesicles, GSTs, and transporters have synergistic effects on anthocyanin sequestration (Zhao, 2015).

Glutathione S-transferases (GSTs) were first found and identified to relieve the toxicity of atrazine, a photosystem II inhibitor (Frear and Swanson, 1970). Subsequent studies found that GST could regulate substance metabolism and participate in biological and abiotic stresses (Edwards et al., 2000; Hayes et al., 2005). Based on the amino acid sequences, GST family was generally divided into different subclasses in Arabidopsis, including phi (GSTF), tau (GSTU), zeta (GSTZ), theta (GSTT), lambda (GSTL), dehydroascorbate reductase (DHAR), and tetrachlorohydroquinone dehalogenase (TCHQD) (Mohsenzadeh et al., 2011). The members of phi subclass are mainly responsible for anthocyanin accumulation (Jiang et al., 2019; Kou et al., 2019; Liu et al., 2019). GST is not an enzyme that directly catalyzes anthocyanin synthesis; rather, it is a carrier responsible for anthocyanins sequestrated in vacuoles (Mueller et al., 2000; Sun et al., 2012). Generally, there is a strong correlation between the subcellular location of GST and flavonoids. In addition, GSTs are relevant for membranes, such as the endoplasmic reticulum (ER) and vacuole, in anthocyanin-rich plant cells (Smith et al., 2003; Sun et al., 2012).

In maize, *Bronze-2(Bz2)* was the first GST gene to be identified to perform an important role in anthocyanin accumulation (Marrs et al., 1995). Subsequently, *Bz2* homologous genes were uncovered in many plants, including Arabidopsis (*TT19*), petunia (*AN9*), grape (*VvGST1* and *VvGST4*), *Camelina sativa* (*CmGSTF12*), sweet potato (*IbGSTF4*), kiwifruit (*AcGST1*), apple (*MdGSTF6*), carrot (*DcGST1*), and cotton (*GhGSTF12*), which implied that the functioning of GST protein in anthocyanin accumulation is highly conserved (Alfenito et al., 1998; Larsen et al., 2003; Kitamura et al., 2010; Wang et al., 2012; Kou et al., 2019; Liu et al., 2019; Meng et al., 2020; Shao et al., 2021). In the Arabidopsis *transparent testa19 (TT19)* mutant, both anthocyanin and proanthocyanidin accumulation in vacuoles of vegetative tissue is affected (Kitamura et al., 2010). Similar to *Bz2* and *AN9*, *TT19* encodes a GST protein, but *AN9* can restore the defective phenotype of the Arabidopsis *tt19* mutant, which cannot accumulate anthocyanin normally (Kitamura et al., 2004). Both *TT19* and *AN9*/*Bz2* may function by stabilizing compounds and sequestrating anthocyanins in the vacuole. The different seed coat color of the Arabidopsis *tt19* mutant may be due to the accumulation of procyanidin precursors in a membrane-encapsulated cytoplasmic structure (Kitamura et al., 2010). Studies have shown that Arabidopsis TT19 protein is located not only in the nucleus and cytoplasm but also in the vacuolar membrane. TT19 protein can bind to anthocyanin monomer without catalyzing the formation of GSH-conjugated anthocyanin *in vitro*. First, TT19 mainly binds to cyanidin on the cytoplasmic surface of the endoplasmic reticulum, yet it also can bind to cyanidin-3-*o*-glucoside in the cytoplasm. Then, the TT19–anthocyanin complex is glycosylated and acetylated in the cytoplasm or on the surface of the vacuolar membrane (Springob et al., 2003; He et al., 2010). Finally, the modified anthocyanins are released from TT19, received by transporters located on the vacuolar membrane, and then finally stored in vacuoles. In addition to TT19 in Arabidopsis, some anthocyanin-related GSTF12 proteins also have been found in various plant species. In red cotton plants, silencing *GhGSTF12* decreased the anthocyanin accumulation but did not affect the expression of anthocyanin biosynthesis-related genes (Shao et al., 2021). *CmGSTF12* in *Camelina sativa* and *PcGSTF12* in pear were identified to affect anthocyanin accumulation in vegetative tissues and proanthocyanidin accumulation in seeds (Wang et al., 2012; Zhang et al., 2020).

Radish (*Raphanus sativus* L., 2n = 18) is an important traditional vegetable in China and cultivated worldwide. The “Yanghua” radish has a characteristic red skin color (i.e., rich in anthocyanins). Some researchers have identified and analyzed the glutathione S-transferase (GST) family in radish but did not study the function of *GST* gene in detail (Gao et al., 2020). In this study, we identified a glutathione S-transferase gene *RsGSTF12* whose function is crucial for anthocyanin sequestration in radish, which provides new insights into the anthocyanin sequestration mechanism in radish.

## Materials and Methods

### Plants and Growth Conditions

The radish cultivar “Yanghua” seeds were sterilized with 8–15% NaClO_4_ for 5–15 min and then soaked in clean water for 6–10 h, after which the seeds were germinated for 30 h under dark conditions. Seedlings with a similar size were selected and planted in pots and grown in 1/4 Hoagland’s nutrient solution for 36 h. Then, all seedlings were moved to lit conditions (light intensity: 200 μmol m^–2^ s^–1^), and samples of them were serially collected at five time points thereafter: 0, 12, 24, 36, and 48 h. Each set of samples were immediately frozen in liquid nitrogen and stored at −80°C for further analyses.

The surface-sterilized seeds of Arabidopsis wild-type Columbia (Col), mutant *tt19*, and transgenic Arabidopsis were vernalized at 4°C for 2–3 days and then transferred to an incubator. To obtain transgenic Arabidopsis seedlings, 15-day-old seedlings were cultivated in nutrition soil. For the experiment investigating RsGSTF12’s function in Arabidopsis, seedlings were cultivated on Murashige and Skoog (MS) medium containing 6% sucrose for 7 days.

### Phylogenetic Tree Analysis of Glutathione S-Transferase Family Genes in Radish

Glutathione S-transferase family member candidates in radish were identified by performing BLASTN searches—under its default algorithm parameters with a cutoff E-value of <10^–10^—of the radish genomic database, using 55 and 88 GST protein sequences, respectively, of Arabidopsis and rice as queries. The identified sequences of RsGSTs, AtGSTs, and OsGSTs were downloaded from the radish genomic database, TAIR database, and MSU Rice Genome Annotation Project, respectively. The sequences of the RsGSTs, AtGSTs, and OsGSTs were aligned by Clustal Omega,^1^ and their phylogenetic tree was constructed using Figtree v 1.4.3 software by the neighbor-joining algorithm. All proteins used in this study are listed in Supplementary Table 1.

### Phenotype and Growth Measurements

The phenotypes of experimental seedlings were recorded with a digital camera (Canon DS126631). Randomly selected seedlings (entire plant and hypocotyl, *n* = 10) were weighed on an electronic balance (Precisa XB 220A, accuracy: ± 0.0001 g).

### Measurement of Total Anthocyanin

The anthocyanin content of the radish and Arabidopsis seedlings was extracted and determined according to previous methods (Su et al., 2014). Briefly, 0.5 g fresh samples (*n* = 3) were thoroughly soaked in 5 mL of methanol with 1% HCL and kept in the dark for 24 h. The absorbance value of each solution was measured by a spectrophotometer (UV-5200 spectrophotometer; Shanghai Metash Instruments Co., Ltd., Shanghai, China) at 530 and 657 nm.

### Extraction of RNA and qRT-PCR Analysis

Total RNA was extracted from “Yanghua” radish seedlings or transgenic Arabidopsis seedlings by using the Omega Plant RNA Kit (CWBIO, CW2598S). First-strand cDNA was synthesized using the reverse transcription kit (Vazyme, R233-01). The primers used for the qRT-PCR were designed in Primer5 software and synthesized by the GENEWIZ Biological Technology Co. (Suzhou, China). These primers are listed in Supplementary Table 2. Full details for running the qRT-PCR can be found in the manufacturer’s instructions of the Bestar^®^ SYBR qPCR Master Kit (Vazyme, MQ101-01). The reaction conditions were as follows: 95°C for 15 s followed by 40 cycles of 95°C for 15 s, 60°C for 60 s, and 72°C for 10 s. A melting curve was produced at the end of each run for each sample. Relative transcript levels were calculated according to the 2^–Δ^Δ*^Ct^* method (Vandesompele et al., 2002).

### Subcellular Localization Analysis

The coding sequence of *RsGSTF12* was PCR-amplified by using its upstream primer and downstream primer (Supplementary Table 2). *RsGSTF12* (without stop codon) was cloned into the pCAMBIA1305-GFP vector to construct the RsGSTF12-GFP recombinant vector. The recombinant vector was transformed into the *Agrobacterium* strain GV3101 with or without tonoplast marker (VAMP711-RFP) and endoplasmic reticulum marker (HDEL-RFP) and then injected into tobacco (*Nicotiana benthamiana*) leaves (Nelson et al., 2007; Sun et al., 2012). Fluorescence was detected under a confocal laser-scanning microscope (Nikon, Tokyo, Japan) at 72 h after the injection.

### Determination of the Ability of *RsGSTF12* in Improving Cyanidin Water Solubility

The *RsGSTF12* or *AtGSTF12* gene was cloned and inserted into the pCzn1 vector between the *Nde*I and *Xba*I sites. Then, the recombinant plasmids pCzn1-RsGSTF12 and pCzn1-AtGSTF12 were each transferred into *Escherichia coli* BL21 cells. Isopropyl-beta-D-thiogalactopyranoside (IPTG) was used to induce the expression of the target protein, and the target protein was re-dissolved by re-denaturation. RsGSTF12 and AtGSTF12 proteins were obtained by nickel (Ni) column affinity purification for use in the subsequent experiments.

The ability of GST proteins RsGSTF12 or AtGSTF12 in improving cyanidin (Cya) water solubility was analyzed by following the methodology of Sun et al. (2012). Briefly, the 1-mL reaction system included 0.1 M KPO_4_ (pH 7.2), 100 μM AtTT19 (or 100 μM RsGSTF12), and 600 μM cyanidin. The mixture was incubated for 120 min, and then, the content of cyanidin in the supernatant was determined by high-performance liquid chromatography (HPLC). Chromatographic conditions of HPLC were as follows: mobile phase A consisted of a 1% acetic acid aqueous solution; mobile phase B of 100% acetonitrile; retain 97% phase A and 3% phase B for 10 min, to balance the column, and the elution procedure lasted 0–1 min (3% B), 1–7 min (50% B), 7–8 min (100% B), and 8–9 min (3% B). The volume size of each sample was 1 μL, with a flow rate set to 1 mL min^–1^. The column temperature was 30°C, and the detection wavelength was 203 and 520 nm.

### Functional Verification of Expressing *RsGSTF12* in Radish Cotyledons

The full coding region of *RsGSTF12* was cloned into the pFGC5941 vector between *Xbo*I and *Xba*I. The recombinant vector was transformed into *Agrobacterium* strain GV3101 *via* the freeze–thaw method. Radish cotyledons were transformed with the pFGC5941 empty vector (i.e., the negative control), the recombinant vector of GmMYB75 (a positive anthocyanin regulator in soybean), the RsGSTF12-5941 recombinant vector, and a mixture of GmMYB75-5941 and RsGSTF12-5941. The phenotypes of radish cotyledons were recorded after injection for 10 days. A confocal laser scanning microscope was used to visualize anthocyanins in the epidermal cells of radish cotyledons (excitation: 559 nm; emission 664 nm). The injected radish cotyledons were collected for their determination of total anthocyanin content and to perform an RT-qPCR analysis. The primers used for this are listed in Supplementary Table 2.

### Constructing the Overexpression and Complement Transgenic Arabidopsis Lines

The coding sequence of *RsGSTF12* was cloned from radish sprouts cDNA, using primers containing *Bam*HI and *Xba*I sites. The ensuing PCR product was inserted into pCAMBIA1305-GFP, and an *RsGSTF12-*1305 recombinant vector was generated, which was then transformed into *Agrobacterium* strain GV3101. The *RsGSTF12-*1305 recombinant vector was transformed into the wild type (WT) and *tt19* mutant by flower dipping (Clough and Bent, 1998). The T_3_ generation was used for further study.

### Statistical Analysis

All experiments were performed at least three replicates, the data of which were analyzed by SPSS 19.0 (SPSS, Chicago, IL, United States). Data are presented as the mean ± standard deviation (SD) whose significant differences are indicated by differing letters according to the ANOVA results (*P* < 0.05).

## Results

### Isolation and Characterization of *RsGSTF12* in Radish

**FIGURE 1:**
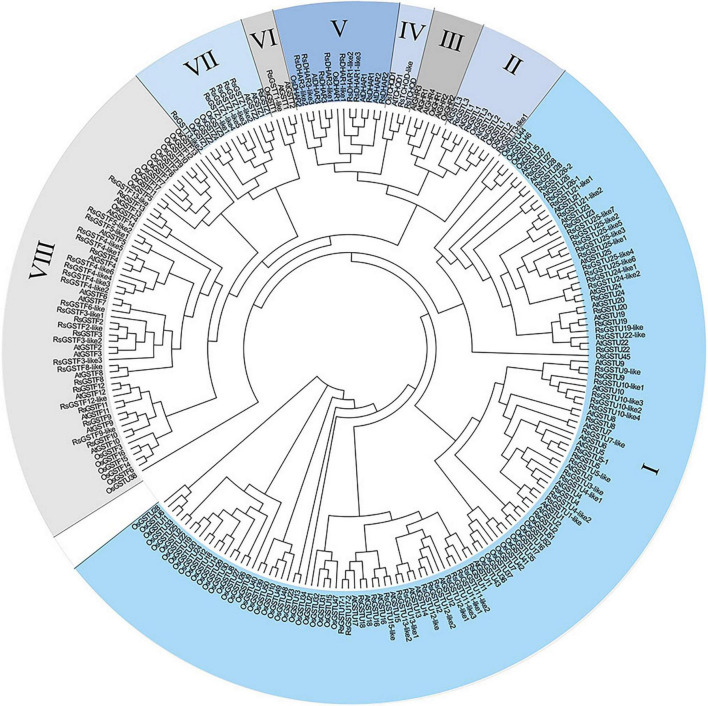
Phylogenetic analysis of glutathione S-transferase (GST) proteins in radish, Arabidopsis, and rice plants. Their protein sequences were aligned with Clustal Omega. The phylogenetic tree was constructed using the neighbor-joining algorithm without distance corrections applied. GST proteins were divided into seven classes. The different colored parts indicate different GST groups. I–VIII represent the tau, lambda, GHR, DHAR, theta, zeta, and phi subfamilies, respectively.

The search for *RsGST* candidate genes in the radish genome was carried out using the NCBI BLASTP, and the protein sequence of each candidate gene was thus obtained. Here, a total of 100 RsGST members were classified into seven major subclasses: *RsGSTU*, *RsGSTF*, *RsGSTT*, *RsGSTZ*, *RsGSTL*, *RsTCHQD*, and *RsDHAR*, respectively (denoting the tau, phi, theta, zeta, lambda, *TCHQD*, and *DHAR* subclasses). Based on their phylogenetic analysis, the RsGSTs were grouped into seven subclasses homologous with Arabidopsis: tau (53 members), phi (26 members), theta (1 member), zeta (6 members), lambda (3 members), TCHQD (2 members), and DHAR (9 members). Among them, the majority of radish GST genes belong to the tau and theta subfamilies, much like the GSTs of other plant species. More importantly, RsGSTF12 is located in the phi subfamily of the GST family (Figure 1).

**FIGURE 2:**
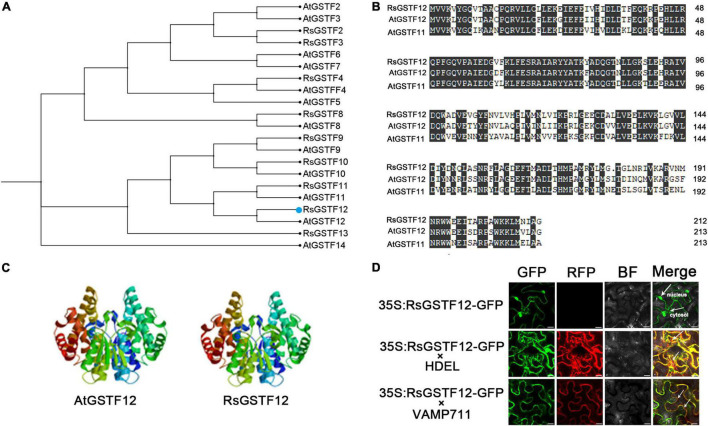
Bioinformatics analysis and subcellular localization of RsGSTF12. **(A)** Phylogenetic tree analysis of RsGSTF12 and some other GSTFs in radish and Arabidopsis. **(B)** Amino acid sequence alignment between RsGSTF12, AtGSTF12, and AtGSTF11. **(C)** The prognostic three-dimensional model predictions of RsGSTF12 and AtGSTF12. **(D)** The subcellular localization of RsGSTF12. Epidermal cells of tobacco leaves expressing RsGSTF12:GFP with or without endoplasmic reticulum-located (HDEL-RFP) and tonoplast-located (VAMP711-RFP) markers, respectively. Scale bar = 20 μm.

We cloned the coding sequence of *RsGSTF12* gene in radish and found that it encodes a polypeptide consisting of 213 amino acids. Phylogenetic tree analysis indicated a high homology of RsGSTF12 with AtGSTF12 (also known as TT19), followed by AtGSTF11 (Figures 1, 2A). The amino acid sequence alignment analysis indicated that RsGSTF12 is highly homologous with AtGSTF12 and AtGSTF11, with all of them having several highly conserved regions (Figure 2B). The three-dimensional structures of RsGSTF12 and AtGSTF12 were predicted by SWISSMODEL online analysis software (Figure 2C). These results showed that the structure of RsGSTF12 was similar to that of AtGSTF12.

To explore the subcellular localization of RsGSTF12, its coding sequence (without the termination codon) was fused with GFP and expressed in tobacco epidermal cells. RsGSTF12-GFP was expressed in tobacco leaves transiently, together with the tonoplast marker and endoplasmic reticulum marker. As shown in Figure 2D, the fluorescence signals of RsGSTF12-GFP were localized in the cytosol and nucleus. Furthermore, RsGSTF12 was co-localized with the endoplasmic reticulum or a tonoplast marker. These results demonstrated that RsGSTF12 was located in both the endoplasmic reticulum and tonoplast.

### *RsGSTF12* Is Strongly Correlated With Anthocyanin Accumulation in Radish Sprouts

**FIGURE 3:**
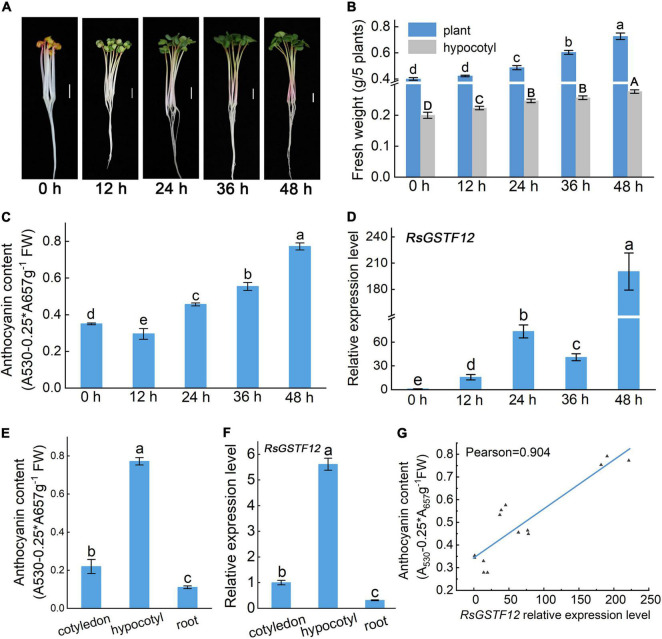
RsGSTF12 has a high correlation with anthocyanin accumulation in radish sprouts. **(A)** Phenotypes of 3-day-old radish seedlings when transferred into a continuous illumination incubator for 0, 12, 24, 36, and 48 h. Scale bar = 1 cm. **(B)** Fresh weight of radish seedlings at the five different time points. **(C)** Anthocyanin content and **(D)** the relative expression levels of *RsGSTF12* in radish hypocotyls at the five time points. **(E)** Anthocyanin content and **(F)** the relative expression level of *RsGSTF12* in different tissues of radish seedlings. **(G)** The correlation between the relative expression level of *RsGSTF12* and anthocyanin in the hypocotyl of radish. Data in panels **(B–F)** are presented as the mean ± standard deviation (SD) whose significant differences are indicated by differing letters according to the Duncan’s multiple range test (*p* < 0.05). The capital and lowercase letters in panel **(B)**, respectively, represent significant differences in hypocotyl and the whole plant weight at different time points.

To observe correlation between the relative expression level of *RsGSTF12* and anthocyanin accumulation, the anthocyanin content in radish hypocotyls was measured at different time points. The fresh weight of both the hypocotyl and the entire plant of radish seedlings increased after they were transferred into the continuous illumination incubator (Figure 3B). Notably, the content of anthocyanin in radish hypocotyl increased with longer illumination time (Figures 3A,C). Interestingly, the content of anthocyanin in the hypocotyl was slightly lower for radish seedlings exposed to light for 12 h than 0 h (Figure 3C). One possible explanation was that age-dependent regulation by the miR156-SPL module might play a predominant role in anthocyanin biosynthesis when radish seedlings were exposed to light for 0 h (Gou et al., 2011). In Figure 3D, the expression level of *RsGSTF12* increased significantly from 0 to 24 h, but then decreased (i.e., it was downregulated); however, at 48 h, it rose again (Figure 3D). The transcription level of *RsGSTF12* presented an upward trend during the growth and development of radish. The *RsGSTF12* transcript abundance was positively correlated with anthocyanin accumulation (Pearson’s *r* coefficient = 0.904) (Figure 3G). In addition, *RsGSTF12* was highly expressed in the hypocotyl of radish seedlings (Figure 3F), in which anthocyanin accumulation was greatest (Figure 3E). Together, these results indicated that *RsGSTF12* was specifically highly expressed in the hypocotyl and *RsGSTF12* was closely associated with anthocyanin accumulation in radish sprouts.

### *RsGSTF12* Increases the Cya Water Solubility

**FIGURE 4:**
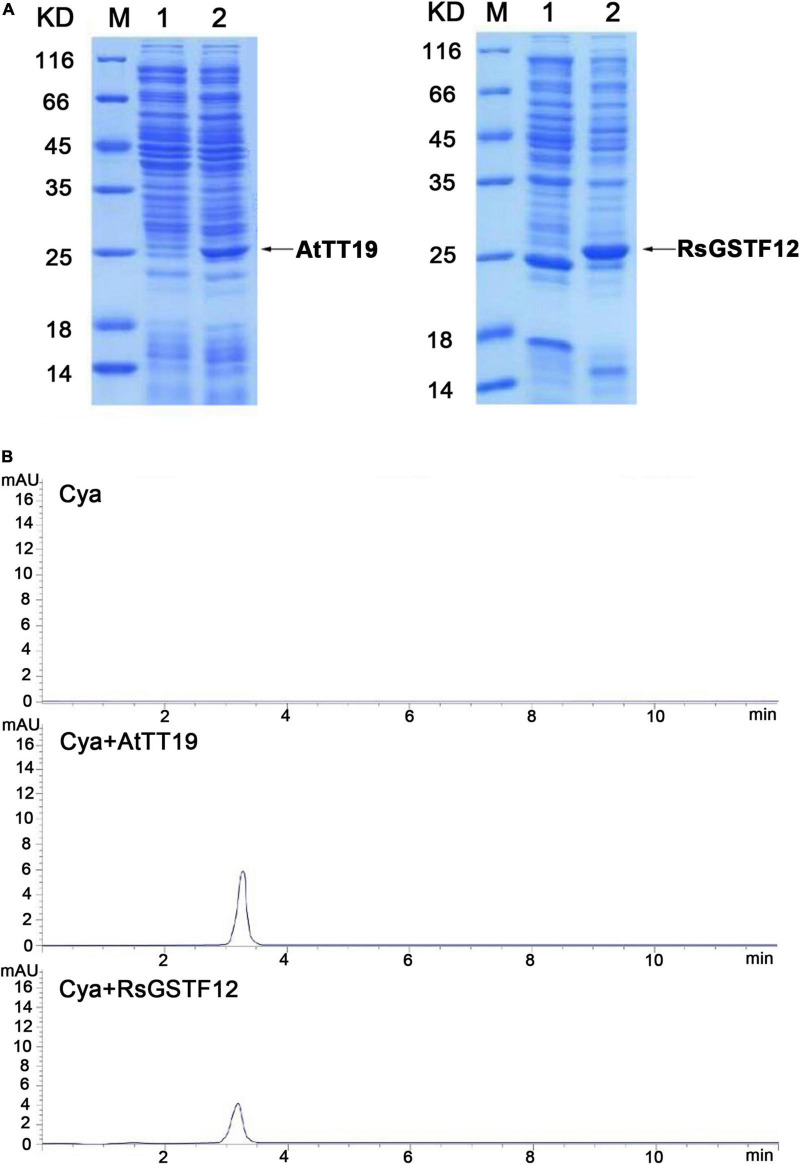
RsGSTF12 combines with cyanidin *in vitro*. **(A)** SDS–PAGE analysis of AtTT19 and RsGSTF12. M, Protein molecular weight marker; 1, uninduced protein; 2, induced protein. **(B)** High-performance liquid chromatography (HPLC) analysis of cyanidin’s content when it was incubated with RsGSTF12 and AtTT19 for 120 min.

To better understand the biochemical properties of RsGSTF12, the CDS of *RsGSTF12* was cloned and inserted into the pCzn1 vector. The empty vector and AtTT19 were considered as negative and positive control, respectively. SDS–PAGE analysis showed that RsGSTF12 and AtTT19 were successfully expressed after being induced by IPTG. The molecular mass of RsGSTF12 and AtTT19 was consistent with their predicted sizes (Figure 4A). AtTT19 in Arabidopsis was reported to have the ability to bind 1-chloro-2, 4-dinitrobenzene (CDNB), which was generally considered as a GSH substrate (Marrs et al., 1995). To investigate whether RsGSTF12 catalyzes the formation of GSH–anthocyanin, the purified proteins were added to Cya solutions and incubated for 120 min at 25°C. The supernatants of three treatments were detected by HPLC, and no peak was detected at 203 nm. The results indicated that RsGSTF12 and AtTT19 could not catalyze glutathionylation of anthocyanin, while remarkable peaks were detected at 520 nm under AtTT19 and RsGSTF12 treatment. As evinced in Figure 4B, Cya could hardly be detected in the supernatant of the control group (Cya) at 120 min, whereas the Cya monomer was detectable in the supernatant of the group treated with AtTT19 or RsGSTF12 protein. In addition, the Cya content was higher under AtTT19 protein than RsGSTF12 treatment. Altogether, these results indicated that RsGSTF12 was capable of improving the water solubility of Cya.

### Expressing *RsGSTF12* Transiently Increases Anthocyanin Accumulation in Radish Cotyledons

**FIGURE 5:**
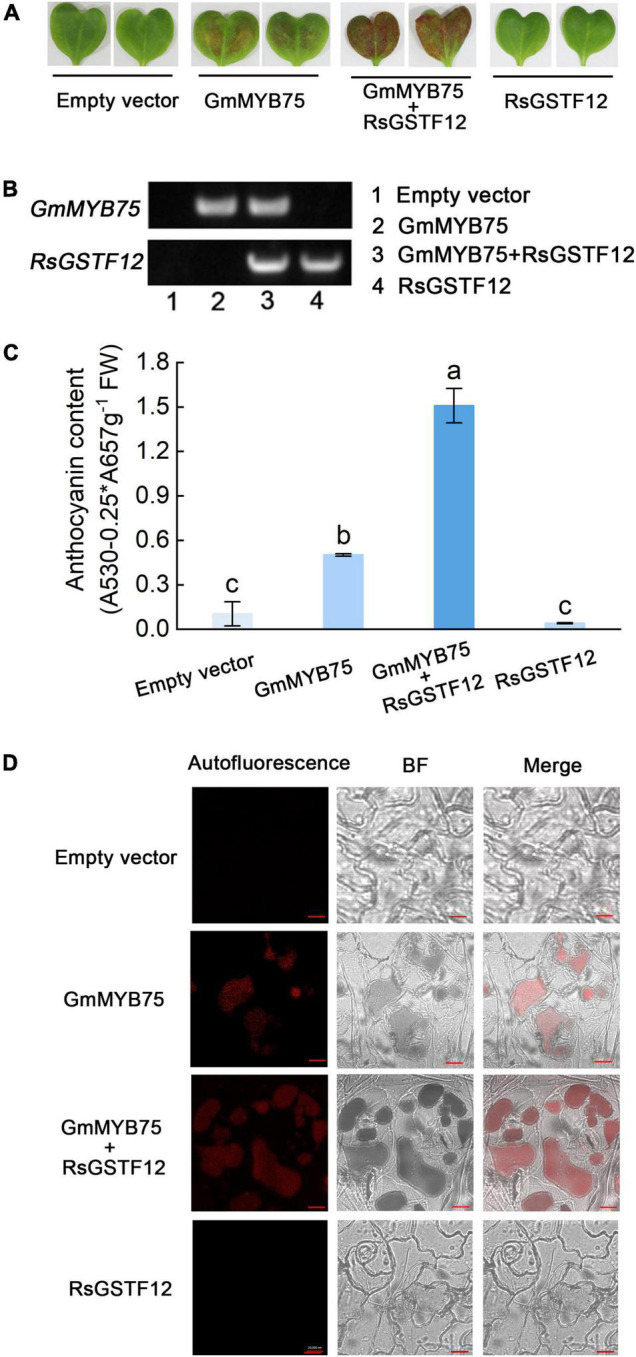
RsGSTF12 increases anthocyanin sequestration in radish cotyledons. **(A)** Phenotypes and **(C)** anthocyanin content of radish cotyledons at 10 days since their injection with the empty vector, GmMYB75, GmMYB75 + RsGSTF12, or RsGSTF12. **(B)** The verification of RsGSTF12 or GmMYB75 was transferred into radish cotyledon. The upper and the lower bands, respectively, represent whether GmMYB75 or RsGSTF12 was transformed in empty vector (1), GmMYB75 (2), GmMYB75 + RsGSTF12 (3), and RsGSTF12 (4) treatments successfully. **(D)** Anthocyanin visualization in epidermal cells of radish cotyledons when expressed with an empty vector, GmMYB75, GmMYB75 + RsGSTF12, or RsGSTF12. Scale bar = 20 nm. Data in panel **(C)** are presented as the mean ± standard deviation (SD) whose significant differences are indicated by differing lowercase letters according to the Duncan’s multiple range test (*p* < 0.05).

To substantiate a role for RsGSTF12 in anthocyanin accumulation, a transient expression assay was performed in radish cotyledons. PCR amplification indicated that both RsGSTF12 and GmMYB75 were transferred into radish cotyledons successfully (Figure 5B). As shown in Figures 5A,C, much more anthocyanin was detected in radish cotyledons when injected with both RsGSTF12-5941 and GmMYB75 (a positive anthocyanin regulator in soybean) than with GmMYB75 alone. However, radish cotyledons injected with RsGSTF12-5941 alone did not undergo anthocyanin accumulation. As evinced in Figure 5C, compared with cotyledons injected with GmMYB75-5941 alone, the red autofluorescence (anthocyanin content) in central vacuoles was more pronounced in cotyledons injected with RsGSTF12-5941 and GmMYB75. Nevertheless, no obvious red autofluorescence appeared in cotyledons injected with either the empty vector or RsGSTF12-5941. Collectively, these results implicated that RsGSTF12 was considered as a prominent regulator of anthocyanin accumulation, in which RsGSTF12 was rendered inoperative in the absence of anthocyanin biosynthesis.

### Expressing *RsGSTF12* Increases the Anthocyanin Accumulation in Arabidopsis

**FIGURE 6:**
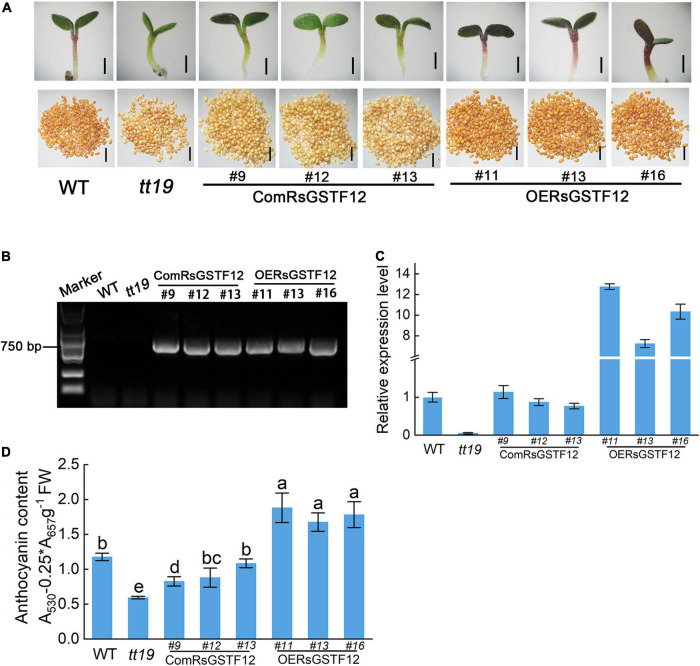
RsGSTF12 increases the anthocyanin accumulation in transgenic Arabidopsis seedlings. **(A)** Phenotypes of the seedlings and seeds of *RsGSTF12* transgenic Arabidopsis. Scale bar = 500 nm. **(B)** Identification of the *RsGSTF12* fragment in *ComRsGSTF12* and *OERsGSTF12* transgenic lines of *Arabidopsis* seedlings. **(C)** The relative expression levels of *AtGSTF12* and *RsGSTF12* in the wild-type, mutant, and transgenic seedlings of Arabidopsis. **(D)** Anthocyanin content in the wild-type, mutant, and transgenic seedlings of Arabidopsis. Seven-day-old seedlings were cultivated on 1/2 Murashige and Skoog (MS) medium with 6% sucrose. Data in panel **(D)** are presented as the mean ± standard deviation (SD) whose significant differences are indicated by differing lowercase letters according to the Duncan’s multiple range test (*p* < 0.05).

RsGSTF12 has a high homology with AtGSTF12 in Arabidopsis, which is essential to anthocyanin accumulation in Arabidopsis (Sun et al., 2012). To investigate the function of RsGSTF12 in the anthocyanin accumulation process, we used the Arabidopsis *tt19* mutant to carry out further research. As expected, the relative expression level of *AtGSTF12* was hardly detected in the *tt19* mutant vis-à-vis the wild-type Arabidopsis (Figure 6C). Then, we transformed 35S:RsGSTF12-GFP recombinant vectors into the Arabidopsis wild-type and *tt19* mutant seedlings, which are named as *OERsGSTF12* and *ComRsGSTF12* transgenic Arabidopsis seedlings, respectively. Finally, we obtained 22 *ComRsGSTF12* and 20 *OERsGSTF12* transgenic lines. Based on the relative expression of *RsGSTF12* in the transgenic Arabidopsis seedlings, the *ComRsGSTF12* transgenic lines (Line 9, Line 12, and Line 13) and *OERsGSTF12* transgenic lines (Line 11, Line 13, and Line 16) were selected for further investigation (Figure 6C). As shown in Figure 6B, RsGSTF12 could be detected by PCR in the *ComRsGSTF12* transgenic lines and the *OERsGSTF12* transgenic lines. As shown in Figure 6A, *ComGSTF12* transgenic seedlings displayed a purple color, whereas the *tt19* mutant seedlings still stayed green at 10 days post-seed germination. Compared with the wild type, the *OEGSTF12* transgenic seedlings featured much purpler. The anthocyanin content of these wild-type, *tt19* mutant, and transgenic lines was consistent with their respective phenotypes (Figure 6D). Compared with *tt19*, anthocyanin content in *ComRsGSTF12* transgenic lines increased by 40.6–83.1%, while when compared with WT, anthocyanin content in *OERsGSTF12* transgenic lines increased by 42.3–59.3%. We also recorded the coloring of the seed coat of the wild-type, *tt19* mutant, and transgenic lines, finding no significant changes when seedlings were transformed with *RsGSTF12*. In summary, the above results provided strong evidence that RsGSTF12 was involved in anthocyanin accumulation.

## Discussion

Previous research has shown that GSTs are essential for the secondary metabolism of plants. Benefiting from the released genomic sequences of radish, here 100 GST candidate genes in this plant were identified, which could be divided into seven distinct subfamilies (Figure 1). Finally, one member, *RsGSTF12*, was chosen among them for further study. By analyzing the phylogenetic tree, we failed to find any GST members in the GHR subfamily, unlike some AtGHRs and OsGHRs, a result consistent with the findings of a recent study (Gao et al., 2020). Not surprisingly, like the GSTs in other plants, the majority of RsGSTs belong to the tau and phi subfamilies, with those GST members responsible for anthocyanin sequestration generally distributed in the phi family (Figure 1; Shao et al., 2021). In addition, RsGSTF12 in radish has a higher homology with AtGSTF12 (AtTT19) (Figures 2A,B). The GSTs in radish and other species (e.g., sweet potato, strawberry, apple, and cotton) all have multiple, highly conserved regions, which suggests that RsGSTF12 has the potential to play a critical role in anthocyanin sequestration (Luo et al., 2018; Jiang et al., 2019; Li et al., 2019; Shao et al., 2021).

Subcellular localization analysis is a valuable method for understanding protein function. Previous research indicated that AtTT19 was localized in the nucleus, cytoplasm, vacuolar membrane, and endoplasmic reticulum membrane and that it can bind with cyanidin (Cya) *in vitro* and improve the water solubility of Cya in Arabidopsis (Sun et al., 2012). In our study, subcellular localization analysis in tobacco revealed that *RsGSTF12* was located in the nucleus and cytosol, and RsGSTF12 could be overlapped with tonoplast and endoplasmic reticulum markers (Figure 2D), which is consistent with the characteristic of AcGST1 in kiwifruit (Liu et al., 2019). GSTs usually exist in plant tissues where anthocyanin accumulates considerably. Disparities in GST localization may arise from using various tissue types of different materials containing varying kinds and concentrations of flavonoids (Zhao, 2015). In radish, *RsGSTF12* was highly expressed in the hypocotyl where abundant anthocyanin accumulated in the peripheral cortex and pericycle (Figures 3E,F; Hara et al., 2003). Further, *RsGSTF12*’s transcript abundance is positively correlated with anthocyanin accumulation (Figure 3G). However, the transcriptional level of *RsGSTF12* was not completely consistent with the anthocyanin content. Whether protein level of RsGSTF12 is highly correlated with anthocyanin content still needs much more attention.

How RsGSTF12 mediates anthocyanin transferred from the endoplasmic reticulum to tonoplast is an important issue to better understand the anthocyanin sequestration mechanism in radish. A kind of GSH substrate [1-chloro-2,4-dinitrobenzene (CDNB)] could be conjugated by GSTs; however, the formation of GSH–anthocyanin has not been identified in plants so far (Marrs et al., 1995). Many efforts have been put in exploring whether GSTs contribute to anthocyanin sequestration by catalyzing the formation of GSH–anthocyanin. For example, AtTT19 in Arabidopsis, AcGST1 in kiwifruit, and ScGST3 in *Senecio cruentus* all did not possess the capability to catalyze the formation of GSH–anthocyanin (Sun et al., 2012; Liu et al., 2019; Cui et al., 2021). In this study, there was no GSH conjugation detected at 203 nm, while remarkable peaks were detected at 520 nm under AtTT19 and RsGSTF12 treatment (Figure 4B). These results indicated that RsGSTF12 could improve the water solubility of Cya *in vitro* to prevent its degradation. In addition, different GSTs are responsible for improving the water solubility of different anthocyanin monomers, which may be because the types of anthocyanin monomers in different species are different (Silva et al., 2016). In Arabidopsis, TT19 increased the water solubility of Cya and cyanidin-3-*O*-glycoside (C3G), while ScGST3 in *Senecio cruentus* was able to increase the water solubility of C3G and delphinidin-3-*O*-glucoside (D3G), rather than Cya and delphinidin (Dp) (Cui et al., 2021). Cya and its derivatives are the main monomers of anthocyanin in radish (Zhang et al., 2019). In this study, we demonstrated that RsGSTF12 may protect the Cya from undergoing degradation so as to improve the water solubility of Cya in solution. Whether RsGSTF12 could improve the water solubility of other anthocyanin monomers still needs further research to explore.

The transient expression system is a direct and important methodological tool used to understand the functions of proteins and is now widely used in various plants, such as apple, pear, and strawberry (Jiang et al., 2019; Castillejo et al., 2020; Zhao et al., 2021). In this study, when RsGSTF12 and GmMYB75 (a positive anthocyanin regulator in soybean) were co-transferred transiently into radish leaves, the leaves appeared much more anthocyanin compared with either of them injected (Figure 5A). Interestingly, the radish leaves injected with RsGSTF12 alone exhibited no significant color change in comparison with the empty vector. Moreover, we observed much more red autofluorescence indicative of anthocyanin content in central vacuoles and vesicle-like structures in those leaves injected with both RsGSTF12 and GmMYB75 vis-à-vis that injected with GmMYB75 or RsGSTF12 only (Figure 5D). One hypothesis to explain this was that when anthocyanin was significantly synthesized, the radish sprouts must also produce more of RsGSTF12 for it to participate in the anthocyanin sequestration process. Recently, new evidence suggested that anthocyanin can be bound and protected by GST, and then, it either was trapped inside the vesicle or formed a complex with the vesicle membrane, to move toward the surroundings of the vacuole (Pourcel et al., 2005; Zhao, 2015). These reports verified our conjecture, to a certain extent, that RsGSTF12 had the potential to be involved in the anthocyanin sequestration. Compared with *tt19* mutant, overexpressing *RsGSTF12* in *tt19* could slightly increase anthocyanin content, while the anthocyanin did not completely recover to the wild-type level (Figure 6A). A similar phenomenon also was observed in maize. ZmGSTIII and Bz2 were both identified as possessing the ability of anthocyanin sequestration, while only knocking out *Bz2* could totally result in anthocyanin accumulation deficiency (Marrs et al., 1995). Possible explanations for this phenomenon were that (i) there exist other GSTs responsible for the sequestration of anthocyanin in radish and (ii) RsGSTF12 has the potential to form homodimer with TT19 (or other GSTs in radish). Loss of TT19 in Arabidopsis led to reductions in anthocyanin and proanthocyanidin in that plant’s tissues and seed coat. Our results showed that the mutant phenotypes of the *tt19* mutant could be partly restored by the expression of *RsGSTF12*. Like the functions of *MdGSTF6* and *GhGSTF12*, *RsGSTF12* could complement anthocyanin pigmentation in vegetative tissues, despite no color change evident in the seed coat (Figure 6) (Jiang et al., 2019; Zhao et al., 2021). Overall, *RsGSTF12* is involved in the process of anthocyanin accumulation.

## Conclusion

In this study, a glutathione S-transferase gene, *RsGSTF12*, was genomically selected that belongs to the phi (F) class. The endoplasmic reticulum- and tonoplast-localized protein RsGSTF12 was highly correlated with anthocyanin accumulation in radish sprouts, which spurred us to explore the functioning of RsGSTF12 in anthocyanin sequestration. Our results confirmed that RsGSTF12 has the ability to improve the water solubility of cyanidin *in vitro*. Furthermore, expressing *RsGSTF12* in radish cotyledons and Arabidopsis seedlings revealed that *RsGSTF12* could promote anthocyanin accumulation. We believe that our research findings may pave a fresh path toward enriching the anthocyanin sequestration network in radish cultivars.

## Data Availability Statement

The datasets presented in this study can be found in online repositories. The names of the repository/repositories and accession number(s) can be found in the article/Supplementary Material.

## Author Contributions

MN, NS, and JCu contributed to the conception of the study. MN conducted the experiments and analyzed the data. MN and CB interpreted the data and wrote the manuscript. JCh, WZ, YZ, and XZ modified the grammar and corrected errors. All authors approved the submitted version.

## Conflict of Interest

The authors declare that the research was conducted in the absence of any commercial or financial relationships that could be construed as a potential conflict of interest.

## Publisher’s Note

All claims expressed in this article are solely those of the authors and do not necessarily represent those of their affiliated organizations, or those of the publisher, the editors and the reviewers. Any product that may be evaluated in this article, or claim that may be made by its manufacturer, is not guaranteed or endorsed by the publisher.

## Footnotes

1
https://www.ebi.ac.uk/Tools/msa/clustalo/
